# “*Bridging the Gap*” Everything that Could Have Been Avoided If We Had Applied Gender Medicine, Pharmacogenetics and Personalized Medicine in the Gender-Omics and Sex-Omics Era

**DOI:** 10.3390/ijms21010296

**Published:** 2019-12-31

**Authors:** Donato Gemmati, Katia Varani, Barbara Bramanti, Roberta Piva, Gloria Bonaccorsi, Alessandro Trentini, Maria Cristina Manfrinato, Veronica Tisato, Alessandra Carè, Tiziana Bellini

**Affiliations:** 1 Department of Biomedical & Specialty Surgical Sciences, and Centre Haemostasis & Thrombosis, University of Ferrara, 44121 Ferrara, Italy; 2 University Center for Studies on Gender Medicine, University of Ferrara, 44121 Ferrara, Italy; katia.varani@unife.it (K.V.); barbara.bramanti@unife.it (B.B.); roberta.piva@unife.it (R.P.); gloria.bonaccorsi@unife.it (G.B.); alessandra.care@iss.it (A.C.); tiziana.bellini@unife.it (T.B.); 3 Department of Medical Sciences, University of Ferrara, 44121 Ferrara, Italy; 4 Department of Biomedical & Specialty Surgical Sciences, University of Ferrara, 44121 Ferrara, Italy; alessandro.trentini@unife.it (A.T.); mmc@unife.it (M.C.M.); 5 Department of Morphology, Surgery and Experimental Medicine, and Menopause and Osteoporosis Centre, University of Ferrara, 44124 Ferrara, Italy; 6 Department of Morphology, Surgery and Experimental Medicine, and LTTA Centre, University of Ferrara, 44121 Ferrara, Italy; veronica.tisato@unife.it; 7 Center for Gender-specific Medicine, Istituto Superiore di Sanita’ (I.S.S.), 00161 Rome, Italy

**Keywords:** gender medicine, sex disparities, genetics/molecular biomarkers, complex diseases, pharmacogenetics, personalized medicine, tailored drug therapy, OMICs, sexomics and genderomics

## Abstract

Gender medicine is the first step of personalized medicine and patient-centred care, an essential development to achieve the standard goal of a holistic approach to patients and diseases. By addressing the interrelation and integration of biological markers (i.e., sex) with indicators of psychological/cultural behaviour (i.e., gender), gender medicine represents the crucial assumption for achieving the personalized health-care required in the third millennium. However, ‘sex’ and ‘gender’ are often misused as synonyms, leading to frequent misunderstandings in those who are not deeply involved in the field. Overall, we have to face the evidence that biological, genetic, epigenetic, psycho-social, cultural, and environmental factors mutually interact in defining sex/gender differences, and at the same time in establishing potential unwanted sex/gender disparities. Prioritizing the role of sex/gender in physiological and pathological processes is crucial in terms of efficient prevention, clinical signs’ identification, prognosis definition, and therapy optimization. In this regard, the omics-approach has become a powerful tool to identify sex/gender-specific disease markers, with potential benefits also in terms of socio-psychological wellbeing for each individual, and cost-effectiveness for National Healthcare systems. “*Being a male or being a female*” is indeed important from a health point of view and it is no longer possible to avoid “*sex and gender lens*” when approaching patients. Accordingly, personalized healthcare must be based on evidence from targeted research studies aimed at understanding how sex and gender influence health across the entire life span. The rapid development of genetic tools in the molecular medicine approaches and their impact in healthcare is an example of highly specialized applications that have moved from specialists to primary care providers (e.g., pharmacogenetic and pharmacogenomic applications in routine medical practice). Gender medicine needs to follow the same path and become an established medical approach. To face the genetic, molecular and pharmacological bases of the existing sex/gender gap by means of omics approaches will pave the way to the discovery and identification of novel drug-targets/therapeutic protocols, personalized laboratory tests and diagnostic procedures (sex/gender-omics). In this scenario, the aim of the present review is not to simply resume the state-of-the-art in the field, rather an opportunity to gain insights into gender medicine, spanning from molecular up to social and psychological stances. The description and critical discussion of some key selected multidisciplinary topics considered as paradigmatic of sex/gender differences and sex/gender inequalities will allow to draft and design strategies useful to fill the existing gap and move forward.

## 1. Introduction

**Figure 1:**
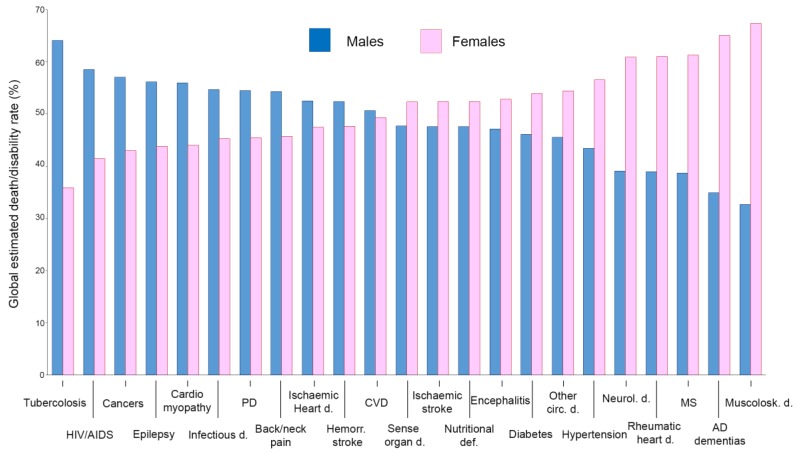
Modified from Global Health Estimated 2016 (www.who.int/healthinfo/global_burden_disease/en/). d.: disease; PD: Parkinson’s disease; Haemorr.: Haemorrhagic; CVD: Cardiovascular Disease; def.: deficiency; circ.: circulatory; neurol.: neurological; MS: Multiple Sclerosis; AD: Alzheimer’s Disease; Musculosk.: musculoskeletal.

The risk of experiencing a particular disease characterized by specific prognosis and outcomes may go behind the natural history of that pathology. It may depend on disparities in prevention, diagnosis and treatment not properly targeted in different gender or sexes, or both. It is to be taken into account that sex has biological features greatly influencing any disease at molecular or epidemiological level, potentially affecting in turn incidence, disease course or treatment output (Figure 1). In May 2014, the US National Institutes of Health (NIH) announced that researchers should account for sex and consider it a biological variable (SABV) in NIH-funded preclinical research [1].

Sex and gender are concepts of primarily relevance in the real world, where disease morbidity, risk factors, onset age, clinical phenotype and treatments may strongly differ between males and females. While sex refers to biological and genetic features, gender includes roles and relationships also related to socio-cultural rules and participation [2,3]. Everybody agrees that “*being a male or being a female*” makes the difference, not only in a medical/healthcare perspective, but also in a social, economic and cultural vision [4].

In the past, medicine was characterized by strong androcentric connotation, and for a long time clinical studies have been mostly performed on male patients, generating results that were merely transferred to females [5,6,7,8]. Accordingly, females were just one third of the enrolled patients in clinical trials performed between 2002 and 2007 to evaluate cardiovascular devices, and unfortunately, the number of recruited females did not increase over time [9]. In another context, it has been demonstrated that the genomic profile of non-small-cell lung cancer patients had strong sex differences in signalling pathways [10], suggesting that prognostic biomarkers could be different and should be selectively used depending on sex. Despite this evidence, the female counterpart itself has been involved in studies restricted to those distinctive branches of female medicine such as gynaecology and reproductive medicine, or particular types of cancer. Finally, male sex also prevails in preclinical studies on animals, creating severe bias on the transposition of results to the clinical practice [11,12,13]. The prejudice leading to the exclusion of female animals was due to their higher biological variability compared to male animals, mainly justified by sex hormone fluctuations. Though, several reports highlighted that data coming from preclinical experimentations based on female animals are useful and reliable [14,15,16]. 

In the recent past, there has been a growing attention to sex-based differences in biology, genetics, biomedical sciences and general medicine, ranging from the cellular level to whole organs and organisms. As expected, this process quickly led to the generation of new insights into diagnostic, prognostic and therapeutic issues, from basic research to the clinical level [17]. The overall message is indeed the one published by *Nature* in 2010, which summarizes old and new problems in the title “Putting gender on the agenda” [18]. Starting from the fact that animals have a sex [19], well known differences in gene expression have to exist in male *versus* female mice [20]. Based on the evidence that companies and scientists may have arbitrarily performed their preclinical tests on male models, the Editors of *Nature* concluded that “Medicine as it is currently applied to women is less evidence-based than that being applied to men” [18]. The increasing attention towards sex and gender, along with the interest that emerges from this kind of aware research, are now beginning to bridge the gap [21]. Thanks to the increased knowledge of the molecular, genetic and epigenetic bases of complex diseases, and thanks to the personalized pharmacogenetic approach to drug design/prescription, several diseases are now faced in a tailored fashion [22,23]. However, while the inclusion of sex is a process already underway, with evident results from both preclinical and clinical trials, the impact of gender in medical/biomedical fields is still at an early stage, with difficulties and delays due to its intrinsic complexity. Ongoing efforts aim to include and understand the role of gender in pharmacology [24,25]. To date, gender-related pharmacodynamic and pharmacokinetic differences have been reported with crucial implications on drugs effects [26,27,28,29,30]. Overall, gender-specific pre-clinical models will increase the definition of gender-oriented therapeutic protocols, in turn accelerating the development of gender-specific drugs and the generation of gender-oriented and evidence-based guidelines [11,31].

Further, omic-sciences offer a useful and powerful analytical option in biomedical research, helping the discovering of novel pharmaceutical targets, bio-molecular markers in a sex/gender-oriented perspective [32,33,34]. Sex and gender, until now merely considered as confounding variables (e.g., “sex and age data adjustment”), are becoming crucial variables in both preclinical and clinical studies. However, while sex has a strong and well defined genetic connotation, the term ‘gender’ shows a broader nuance with different meanings in biomedical and social sciences and is often used improperly as a synonymous of ‘sex’ [35,36]. ‘Sex’ and ‘gender’ are no longer synonyms, considering that cell lines have one sex, preclinical models have one sex, whilst humans have both gender and sex. 

In this regard, it should be emphasized the role that the European Union (EU) has in supporting targeted projects and actions. Among these, the GenderBasic Project was created to promote gender integration in basic research [37,38], whereas the EUGIM Project to establish a European Curriculum in gender medicine. In the field of cardiovascular disease, the EUGeneHeart Project was generated to develop new approaches for prevention and treatment of heart failure through the analysis of genomic signalling [39], while the GenCAD Project aimed to improve the knowledge on sex and gender differences in cardiovascular and other chronic diseases [40]. More recently, the European Network for Gender Medicine (EUGenMed, EUGenNet) has developed a roadmap for implementing sex and gender concepts in biomedical and health research [21,41,42,43]. Other actions designed to contribute to gender equality have also been implemented, including the establishment of the European Institute for Gender Equality (EIGE, [44]).

For this review, we selected paradigmatic medical issues, in which sex is a determining factor in symptoms, outcome, treatment efficacy, prognosis or epidemiology, to identify different features not properly considered to date, and find possible candidates to overcome the sexual gap we have created in the past. Specifically, the most representative gaps and differences that best characterize the single disease or a group of them from a biological, genetic, molecular or epidemiological point of view, and that may influence the incidence, the course of the disease or the treatment output are addressed. Overall, we critically discuss *facts and mistakes* with the aim of verifying *whether and what* we have learned from the past, and of filling the gap in the light of an emerging new personalized sex/gender-omics medicine.

## 2. Sex Disparity in Cardiovascular Disease

Among cardiovascular diseases, acute myocardial infarction (AMI) shows significant differences in occurrence rate, prognosis and efficacy of treatment between male and female patients. Improvement in the acute treatment has led to a dramatic increasing in the number of AMI survivors among those with damaged heart. These patients are at risk of developing severe complications, like left ventricular remodelling (LVR) and heart failure (HF), which are still considered orphans of specific prognostic tools and effective dedicated treatments [45,46]. AMI is the most frequent cause of LVR and HF and patients often undergo to chronic and costly therapies, very frequent re-hospitalization and poor quality of life, with a significant gap among the two sexes [9,45,46]. Recently it has been reported that “the incidence and prevalence of coronary artery disease in women has exceeded that in men over the past four decades” [47,48].

AMI takes place from a complex interrelationship among genetic/epigenetic and environmental risk factors, as it was revealed by several studies of pharmacogenetics and -genomics [49,50,51], and more extensively confirmed by omics investigations [32,33]. In addition, a very recent study from the Multi-Analyte, Genetic, and Thrombogenic Markers of Atherosclerosis group (MAGMA) found that females with angina are more thrombogenic than males, and this difference may affect sex-related outcomes [52], also on the basis of undeniable genomics differences. Moreover, the Genetics of Subsequent Coronary Heart Disease consortium (GENIUS-CHD) was established to discover and validate genetic variants and biomarkers for the risk assessment of subsequent ischemic events along with novel drug targets for secondary prevention. It is considered a first-class initiative able to generate extraordinary results also in the field of sex-oriented cardiology [53]. Our group has contributed for a long time to the disclosure of the genetics/pharmacogenetics bases of myocardial infarction [54,55,56,57,58,59], and very recently we summarized our previous efforts and patents [US2016363592 (A1); ITTO20130532 (A1)] in a Special Issue belonging to the “Novel Molecular Targets for Cardioprotection: The EU-Cardioprotection Cost Action (CA16225)”, which suggest useful sex-oriented prognostic biomarkers [60].

**Figure 2:**
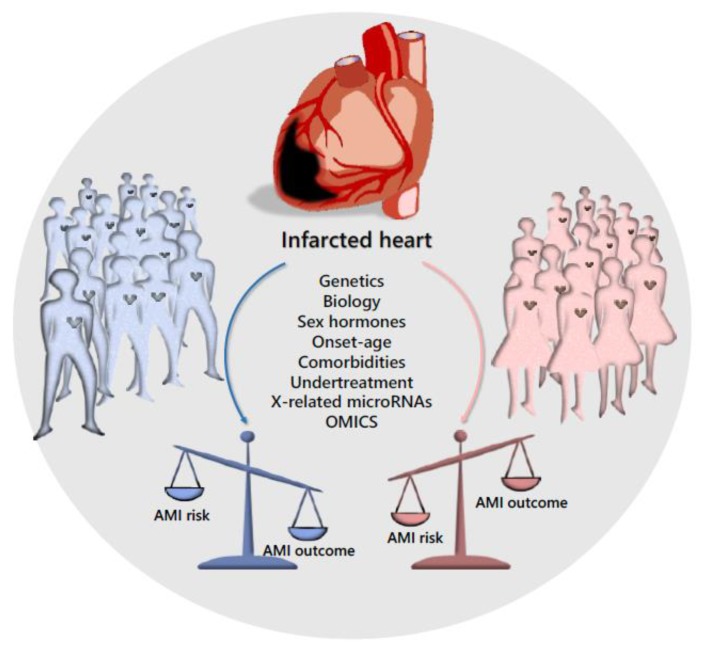
Extreme clinical phenotypes and prognosis in male and female AMI patients. The increased AMI risk in males is balanced by a better prognosis, resulting in enhanced AMI outcome. Conversely, the reduced AMI risk in females is characterized by a worst prognosis, resulting in a poor AMI outcome.

Noteworthy, the incidence of AMI is much lower among females under the age of 50 years compared with males, but after the menopause, the rate in females dramatically increases, approaching that of males [9,61]. For this reason, oestrogens were postulated to be cardioprotective but results coming from recent randomized clinical trials challenge this hypothesis [61]. In addition, sex differences influence AMI pathophysiology, clinical presentation and clinical outcome. In detail, the mortality rate after one or five years is higher in females than in males, and the former more frequently experience heart wall remodelling, failure and stroke within five years from the first ischemic accident. This remains the poorest outcome, even considering several concomitant situations, like different age at presentation, genetic risk factors and comorbidities, as demonstrated by the higher in-hospital mortality, the readmission rate within the first thirty days or the longer follow-up [9]. Interestingly, results coming from five Italian prospective registries conducted between 2001 and 2014, concluded that age and sex specific differences exist in the outcome of patients with ST-elevation myocardial infarction (STEMI) [61,62]. Regardless of age, at discharge, females are at higher risk of re-hospitalization due to different causes. Strong disparities between the two sexes were confirmed also after adjustment for confounding factors [63]. Accordingly, the in-hospital death was 3.2% for males and 8.4% for females, and the latter have been found significantly associated with in-hospital mortality. Finally, females continue to experience higher post-AMI mortality and global poorer outcome (Figure 2) also despite the improvements in reperfusion therapy tools [64]. In conclusion, despite a worldwide improvement in STEMI care, females continue to experience higher in-hospital mortality, which is not completely unexpected, as the most fundamental cardiovascular studies are primarily based on males [9,65].

Another key element is that postmenopausal females tend to develop HF with preserved ejection fraction, characterized by diastolic dysfunction, whereas age-matching males develop HF with reduced ejection fraction, characterized by systolic dysfunction [66]. These differences are important because most drugs used to treat HF have been developed to treat those with reduced ejection fraction, and there are few effective treatment options for HF with preserved ejection fraction [67]. It is also possible that the aging process affects the heart of males and females differently, so that the latter are predisposed to problems of myocardial relaxation, while males mainly develop pump failure. As a result, males and females show a completely opposite trend in terms of hospitalization index for HF, which increases significantly with the age of the females compared with age-matching males [68]. A recent long-term study on LVR after the first AMI reports that, of the total number of patients who experienced LVR after one-year follow-up (31–38% in the two cohorts of the study), females were significantly over-represented (43–46.5% vs 27–36.5% for females and males respectively in the two studies) [69]. These data are extremely important since both cohorts of patients in that study had a very high rate of secondary prevention medications after one year (i.e., beta-blockers 90–95% and ACE inhibitors/angiotensin II R-blockers 93–97% respectively in the two studies). Interestingly, sex hormones are associated differently with the morphology of the remodelled heart wall, and a more androgenic profile is responsible for a greater imbalance in the heart mass/volume ratio in females than in males [70].

In summary, remodelling is the main determinant of survival after recovery from AMI. Observational clinical and post-mortem/experimental studies suggest important differences between females and males in the cardiac remodelling observed in response to different types of cardiac injuries including infarction. Recommended therapies for AMI in females are similar to those in males, but studies indicate that females are undertreated, which lead to worse outcomes [9,47]. Interestingly, during the first few hours after AMI, complex local healing processes and inflammatory reactions are crucial in determining the risk of wall remodelling. Soluble molecules, resident and circulating cells and micro-RNAs finely mediate inflammation and regenerative progression by determining the fate of the heart after the infarction [71,72,73,74]. Basically, imbalance in any healing process and unrestrained ECM proteolysis cause a delayed remedy, while lasting chronic lesions in any district may affect the normal organ/tissue functions with consequent failure and malfunction [75,76,77]. Since these complex processes are in part genetically determined, molecular regenerative medicine, by providing molecular predictive indicators, might help clinicians to recognize at risk populations also in a sex- or gender-oriented direction [78,79,80,81]. Reperfusion therapy and systematic use of evidence-based medications have effects on LVR or HF but they cannot be considered dedicated therapeutic approaches. Prognostic biomarkers able to score cases at higher risk to develop severe post-infarction complications (i.e., females) will allow the early identification of patients for whom the available standard care is not adequate. It is to be taken into account that groups of males at higher risk could also benefit from personalized approaches.

Overall, there is a mandatory need for a “holistic” sex specific approach (i.e., sex-oriented omics-investigations “sex/genderomics”), from the use of in vitro and in vivo preclinical models up to appropriate clinical studies with well-balanced male/female ratio cohorts. This will allow the identification of prognostic biomarkers to effectively counteract sex-disparities and help in designing sex-dedicated therapies [82]. If appropriately addressed, these approaches will represent a significant improvement in the cardiovascular disease management, and the potential of sex-specific risk stratification tools will positively influence the worldwide National Health Care Systems.

## 3. Sex Disparity in Cancer 

Although in the last decades very important results based on novel therapeutic approaches have been obtained, cancer is still a major cause of death today, with an increasing incidence worldwide [83]. It is well known that on average men live less than women (in Italy the average age is 80.8 and 85.2 years, respectively, according to data from the National Institute of Statistics), and this difference is even greater in populations with a higher life expectancy [84].

Sex- and/or gender-associated differences in cancer incidence, prognosis, response to therapy and, eventually, survival have been fully reported with epidemiological data that show, with few exceptions (i.e., thyroid carcinoma), a general female advantage [85]. According to the Italian Cancer Registry (AIRTUM), one in two men and one in three women have an average lifetime risk of developing cancer, while one in three men and one in six women a mean probability of dying from cancer. However, at present, few data exist on the mechanisms underlying these disparities. Among the key factors, we primarily should consider the roles of sex chromosomes, sex hormones and immune responses.

Looking at differences between female XX and male XY cells, evidence based data showed that female cells have higher capabilities to overcome cellular stress through the induction of protective mechanisms, like autophagy, and more antioxidant defenses than male cells [86]. In addition, the random inactivation of one X-chromosome in each single female cell leads to mosaicism and in turn to the advantages associated with genetic heterogeneity. Theoretically, inactivation should balance the expression of X-linked genes between men and women; practically it is incomplete with a significant amount of genes escaping this process to rate greater than 15% of the total. In fact, the presence of mutations in tumour suppressor genes on a single allele, by retaining two functional copies, might represent a protective mechanism [87]. It is important to note that the X-chromosome is significantly enriched for immune-related microRNAs whose deregulation has been associated with the pathogenesis of many kinds of cancers. Recent data reported the identification of nearly 120 microRNAs on the X-chromosome, in contrast to the four found on the Y-chromosome, whereas autosomes on average contain 40-50 of them. These small non coding RNAs (20–25 nt long), acting as post-transcriptional regulators of the gene expression, represent a really powerful regulatory system. Since the X-chromosome is enriched also for immune-related coding genes, the option of sex-associated functional loops can also be hypothesized [88].

Considering the role of sex hormones, oestrogens and androgens have been shown to modulate immune responses, resulting in a different gender susceptibility to diseases [89]. Indeed, female immune functions and responses are generally higher than in males, on one side sustaining a stronger immune response against infections, on the other increasing susceptibility to develop autoimmune diseases [90]. Many important examples of cancer-associated gender differences have been reported, and among them we can highlight colorectal and bladder cancer as well as melanoma [91,92]. Colorectal cancer, the third most common cancer in the world, is characterized by sex- and gender-specific differences, since women appear more prone than men to develop right-sided colon cancer, a more aggressive form of this neoplasia. Interestingly, right and left localized tumours are associated with different molecular abnormalities, i.e., microsatellite instability (MSI) and *BRAF* mutations are often observed in right-sided colon cancer, whereas chromosomal instability and p53 mutations are more frequent in left-sided tumours. Besides anatomic and physiological differences of the colon (longer transverse colon in women), hormonal factors might underlie the observed differences, since oestrogen appears to be a protective factor against MSI, as suggested by the increased risk of MSI-high colon cancer in older women and by the reduced risk in postmenopausal women undergoing hormone replacement therapy [93,94]. In addition, socio-cultural disparities, as dietary factors, should be considered.

**Figure 3:**
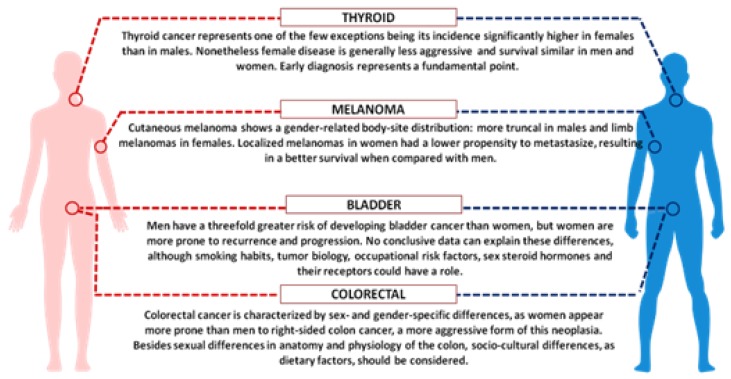
Schematic picture showing key examples of sex and gender disparities in cancer.

Gender discrepancy also exists in the incidence of bladder carcinoma, the fourth most common cancer in men and the seventeenth in women worldwide (Figure 3). However, women are more prone to both recurrence and progression of the disease. One explanation could rely on differences of female and male anatomic sites: the higher frequency of infections (e.g., cystitis) in women might cause delayed diagnoses with negative effects on prognosis and quality of life. In addition, gender-associated specificities, such as smoking habits and occupational risk factors, may play a role [95].

Finally, we should focus on cutaneous melanoma, which show better results in women compared with men [96]. Although male/female incidence ratios vary widely across continents, the female survival advantage has been reported very consistently everywhere and gender remains an independent prognostic indicator after adjustment for thickness and body sites. Differences in detection might be explained by the known gender differences in the body-site distribution: more truncal melanomas in males and limb localization in females. Furthermore, men are less likely to engage in preventive actions. Since cancer is the result of failed immune surveillance, the divergent effects of male and female sex hormones on anticancer immunity could contribute to the higher cancer incidence and poorer outcome in men, particularly in highly antigenic tumours like melanoma [97]. In recent years, several immunological therapies have been approved for different types of tumour, initially based on blocking antibodies against the programmed death receptor-1 (PD1) or its ligand PDL1. These target molecules are expressed on T-lymphocytes and on tumour cells and the receptor-ligand binding interferes with T cell-mediated responses. Treatments with immune checkpoint inhibitors (now developed against many other surface markers), impairing the receptor-ligand binding and the consequent inhibitory response, promote the T-cell activation. A number of meta-analyses, run to evaluate the efficacy of these inhibitors, showed a certain degree of heterogeneity between men and women. The immune checkpoint inhibitors can improve the overall survival for patients with advanced cancers, particularly melanoma and non-small-cell lung cancer, and the extent of the benefit appears sex-dependent. Unexpectedly, despite the overall strong female immune responses, the results derived from clinical trials indicated a smaller benefit for women [98,99]. A review paper analysing over 11,000 patients treated with immune checkpoint inhibitors (i.e., ipilimumab, tremelimumab, nivolumab, or pembrolizumab) in twenty randomized controlled trials, evidenced that the overall survival was consistently higher for men than for women, regardless of cancer histotype, line of treatment and type of administered drug [99]. Further analysis, focused on phase III RCTs of ICIs efficacy in advanced cancers, confirmed the more favourable outcomes in men than in women, particularly with anti-CTLA-4 agents [100]. Even though the biological evidences behind the different efficacy in the two sexes are still lacking, we could suppose that the female immune system has *per se* a strong effect in determining the anti-CTLA4 and anti-PD1 efficacy, thus possibly limiting the effect of other variables. Although we could note that the expression of PD-L1 appears to be directly or indirectly controlled by several X-linked microRNAs [101], the suggested role of PD-L1 expression level as a predictive biomarker of efficacy is quite controversial [102].

In conclusion, it is absolutely relevant that preclinical studies use animals of both sexes to investigate the molecular mechanisms underlying cancer development and progression. Further, sex and gender should be considered in clinical trials for more accurate diagnosis, correct stratification of patients and proper therapies. In the era of precision medicine, the goal will be to identify molecular drivers, possibly different in males and females, to predict responders and non-responders and select the best therapeutic action for each one. According to the recently approved Italian law 3/2018, for “Diffusion and Application of Gender-specific Medicine in the National Health Service”, sex and/or gender should be included in all the health care aspects, hopefully through new specific guidelines.

## 4. Sex Disparity in Neurodegenerative Disorders

Sex differences exist in neurological and neurodegenerative diseases, and epidemiological studies clearly indicate that both frequency and symptoms presentation have a dimorphic behaviour. The most evident examples are represented by multiple sclerosis (MS), Alzheimer’s disease (AD) and Parkinson’s disease, the first two with a higher prevalence in females, the latter with a higher frequency in males [103]. It has been established that sex differences start during neurodevelopment and continue throughout the growth, affecting brain morphology and neuronal connectivity [104]. As such, sex differences may also reveal a differential vulnerability towards neurodegenerative disorders, thus providing the premises for the observed sex-based unbalanced frequency and severity. Neurological and neurodegenerative disorders are characterised by different clinical phenotypes, pathogenesis, clinical onset and progression, they nonetheless share common patterns like the role of specific genetic factors, the impact of gonadal hormones and the role of neuro-inflammatory processes/mediators. Here, we will report on the progression of sex-oriented studies on the two major neurological disorders, MS and AD.

### 4.1. Sex Disparity in Multiple Sclerosis

**Figure 4:**
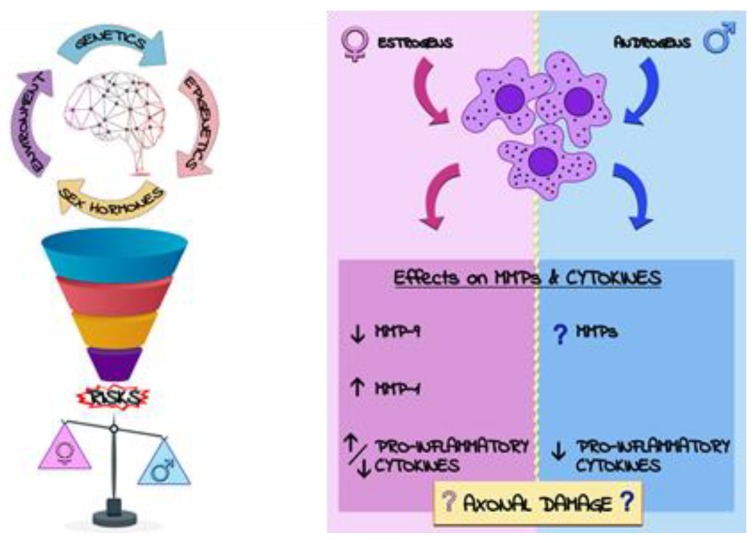
Factors concurring to different MS risk in females with respect to males. Genetics (e.g., HLA-DRB1*1501 allele), epigenetics (e.g., TLR7, CD40L, FoxP3), environment (e.g., smoking and Vitamin D deficiency) and sex hormones (e.g., oestradiol, progesterone, oestradiol and testosterone).

MS can be considered a prototypical sex-dimorphic neurodegenerative disease where (mainly unknown) genetic and epigenetic factors may interact with environment and sex hormones to increase disease susceptibility and progression (Figure 4) [105]. MS is a chronic inflammatory disease of the central nervous system (CNS) with a supposed autoimmune base and unknown aetiology characterized, in its more frequent form, by relapsing-remitting (RR) attacks [106]. These attacks represent a sequence of events of inflammation, demyelination and axonal damage that recur over time and lead to variable spectra of neurological symptoms and signs [107]. International literature confirms the evidence that MS is more frequent in females than in males with a ratio of nearly 3:1 and an earlier onset of the disease, although males generally have a more progressive course and greater disability [104].

The role of sex hormones in MS has been underlined in several studies, in which the most evident example is the so called “pregnancy effect”. Although the mechanisms are still far from clear, it has been observed that the symptoms decrease during pregnancy, in correlation with increased levels of progesterone, oestradiol and estriol, thus returning to the previous stage of the disease or even to a worse stage after delivery, when the hormones revert to the basal condition [108]. This is not surprising since several observations confirmed the modulatory actions of sex hormones on immune cells [104,105], which is of paramount importance considering their role in MS pathogenesis. Indeed, immune cells mediate both neuro-inflammation and partly axonal damage, the two main disease hallmarks leading to disease progression and increased patients’ disability. Basically, MS patients exhibit an impaired Blood Brain Barrier (BBB), which indicates infiltration of leukocyte into the CNS, reflecting in increased circulating levels of active Matrix Metalloproteinases (MMPs) [109,110,111]. The same is true for pro-inflammatory cytokines, which are found to be deranged and increased both in blood and cerebrospinal fluid (CSF) of MS patients compared to controls [112,113]. Similarly, biomarkers of axonal damage, in particular the neurofilament light subunit (NfL), are significantly higher in patients with MS and other neurodegenerative disorders [114,115,116].

Despite the evident importance of the aforementioned biomarkers in MS physiopathology, the possible dualistic influence of sex on the MS features in relation to their levels is still largely unexplored. This is particularly evident for MMPs, for which the paucity of in vivo studies have only examined cohorts of patients without MS, despite the undisputed importance of these enzymes in the disease [110]. The few studies on MS reported an upregulation of MMP-9 in peripheral blood mononuclear cells (PBMCs) of females with the RR form [117] and a decreased expression of MMP-9 following treatment with estriol in the preclinical model of the disease [118]. Currently, there is only one in vivo study showing for the first time increased cerebrospinal fluid (CSF) concentration of MMP-1 in females with MS [119]. Because of the paucity of data, it is evident that several gaps and controversies still exist in literature; therefore, more effort should be put in this field in order to discover novel sex-specific biomarker patterns related to MS. Nonetheless, the few in vitro studies conducted so far suggest that estriol, and in general female hormones, modulates MMPs more likely through an indirect interference with downstream cascades (e.g., by activating/inhibition NfKB, AP-1 and STAT transcription factors) mediated by interleukins (ILs) [120]. Contrariwise, information about a possible influence of androgens on MMPs production from immune cells is still lacking. The aforementioned connection is all the more important considering the involvement of cytokines derangement in MS. Indeed, several works suggest that both female and male sex hormones have tremendous effects on the response and development of peripheral immune cells, depending on the type of receptor stimulated and the origin of cells. For instance, a low concentration of the main oestrogen 17β-estradiol (E2) seems to promote pro-inflammatory response. However, a high or supra-physiological concentration of oestrogens inhibits pro-inflammatory responses, an effect that is probably mediated by interferences with collateral signalling cascades activated by the receptor [121]. Of note, the effect of oestrogens on immune cells is complex since their actions could be mediated by oestrogen receptors (ER) ERα and ERβ, showing different outcomes based on the one stimulated [122]. Conversely, androgens seem to play a positive role in the development and function of the innate immune cells, with a general inhibitory action on adaptive immune cells [123]. Finally, androgens have been shown to inhibit the Th1 differentiation of CD4+ cells, a type of cells strictly related to MS [121]. These evidences were also translated to MS with a pilot study showing that the treatment of PBMCs with estriol shifted the T-cell phenotype from a Th1 profile towards a more predominant Th2 phenotype [124]. This was not the only observation of a sex bias on the cytokine levels in MS. Other studies found an impaired response of females to myelin peptides (proteolipid protein peptides, PLP), with a stronger release of IFN-γ but no secretion of IL-5 when compared to males, suggesting a skewed Th1 phenotype [125]. However, other studies found contrasting results, reporting no significant sex differences in some cytokines (IFN-γ, IL-2, TNF-α, IL-4, IL-10, IL-13). Though, females demonstrated a more predominant pro-inflammatory profile [126,127]. In addition, changes in secretion of cytokines were found to be disease-phase dependent and affected by sex: female patients compared to males have higher pro-inflammatory cytokine levels in the progressive phase and lower levels in the relapsing phase [126]. Taken together, these evidences confirm that the immune system is strongly influenced by sex and may play an important role in determining the “gender gap” characteristic of MS, although the cause is more likely to be multifactorial [128].

Finally, although still in its infancy, several lines of evidence are accumulating on the sex-based differences in axonal damage biomarkers in MS, particularly for the neurofilament light chain (NfL). Indeed, the most recent data coming from a meta-analysis published in 2019 [116], which included more than 1600 MS patients (both RR and progressive), found that males had higher CSF levels of NfL than females, an observation that was also reported for other neurological conditions like Alzheimer’s disease [129]. Other relatively small single centre studies were unable to confirm the sex influence on NfL concentrations [130,131], yet some of these studies measured NfL in serum not considering CSF. Although the employed assays are virtually the same and it has been reported a strong correlation between serum and CSF NfL levels [132], we cannot rule out that possible sex-related differences may primarily exist in brain with higher effects on CSF rather than at systemic level. In addition, the reduced sample size may also have weakened the statistical power to identify the rather small effect size of sex (CSF male/female ratio for NfL mean values in untreated RR patients: 1.16; 95% CI, 1.06-1.27) [116]. Nonetheless, the higher NfL values in male MS patients might in part explain why they are characterized by more aggressive progression of disease and symptoms compared to females, suggesting that the axonal damage may be more prominent in males. However, the same pattern observed in healthy males may indicate that this peculiar characteristic is not specific to the disease, but rather a sex-based biological feature [116]. Finally, brain iron homeostasis is known to be disturbed in MS, and several genetic studies confirmed different distributions of common gene variant in MS subtypes and in sex [133,134]. Rare variants and variations in copy number were also detected [135,136], as well as altered microRNA expression or dysregulated RNA splicing [137,138,139].

In conclusion, in vitro studies to explain the possible molecular mechanisms responsible for the influence of sex hormone on axonal damage and biomarkers are still lacking. Our analysis clearly highlights the existence of a knowledge gap responsible for the prevailing disparities in neurodegenerative disorders. Accordingly, the scientific community is strongly pursuing this task in order to achieve the ultimate goal of dedicated and personalized diagnosis and treatment of MS.

### 4.2. Sex Disparity in Alzheimer’s Disease

The worldwide picture of dementia is dramatically impressive, reporting about 50 million people with cognitive and related functional disability which results in the fifth leading cause of death, according to data of WHO [140]. It has been estimated that more than one hundred million people will globally experience dementia in 2050, with massive social and economic impact that asks for straightforward interventions aimed at reducing this picture by improving and optimizing earlier diagnosis and personalized treatments [140,141]. Alzheimer’s disease (AD) is a multifactorial neurodegenerative disease and it represents the main form of dementia affecting patients, accounting for up to 80% of all cases in which sex, genetic, intellectual, as well as psychosocial factors might play a role in favouring cognitive decline [142]. Of note, genetic susceptibility and intellectual/social/ psychosocial dynamics may differentially affect the cognitive decline onset and progression, with crucial effects on diagnosis time, prognosis and efficacy of therapeutic approaches [143]. Overall, epidemiological studies highlight that females are at greater risk of dementia compared to males, with a two-fold higher risk of AD [144,145,146], and although the risk of vascular dementia (the second most common type of dementia accounting for 15–20% of cases [147]) is lower than in males, females experience a more severe clinical phenotype [148]. The higher life-expectancy of females compared to males makes nonetheless difficult to find clear associations with the increased risk experienced by females, that may also be affected by additional factors like access to education and social or economic status [143].

**Figure 5:**
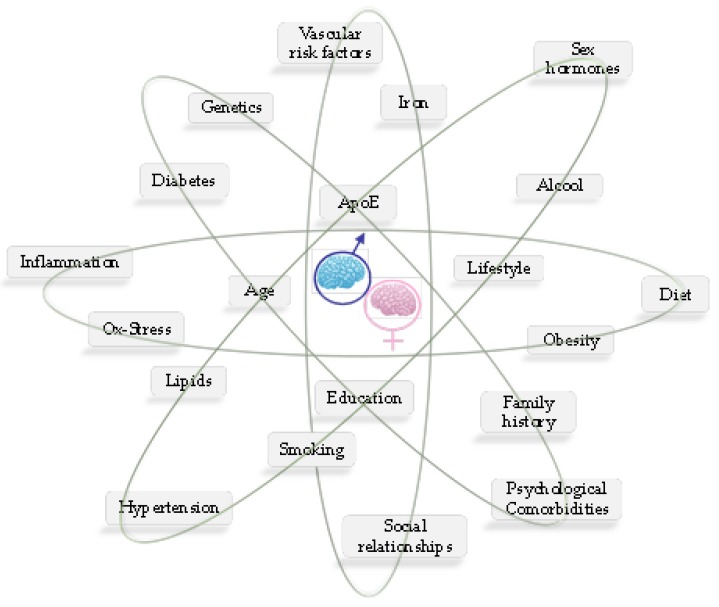
Snapshot of key risk factors for the development of AD and other types of dementia.

Several genetic contributors to the AD risk have been identified, including gene mutations, splice variants, or single nucleotide polymorphisms (SNPs) [149]. Among the strongest genetic factor involved in AD, the ɛ4 allele of the Apolipoprotein E gene (*APOE*), has been deeply investigated and it is now widely recognized as the strongest genetic factor in determining AD risk in the common late-onset form [150]. Its involvement in other forms of dementia is now also emerging [151,152,153,154]. Subjects carrying the *APOE*-ε4 allele are at higher risk in the overall population, while sex specific analyses showed that females experience a more severe clinical phenotype and are at higher risk to convert and progress from mild cognitive impairment towards the more severe AD [155,156]. Similarly, variants of the oestrogen receptor α-gene (ESR1) have been found involved in sex-specific delay of the onset of AD in females [157]. Gene-gene and gene-environment interactions in AD significantly contribute to the different risk in specific groups of patients. In this respect, AD and other types of cognitive impairment share the imbalance/alteration of the homeostasis of iron and lipids involved in the reported sex-differences. Iron and cholesterol accumulation has a strong influence on AD. Accordingly, associated gene variants might be appealing candidate for risk and disease progression assessment. In this scenario, our group recently demonstrated that established genetic risk factors, like the different *APOE*-alleles, might be affected by key genetic backgrounds, making patients differently able to manage local iron accumulation [158] (Figure 5). Studies focused on the link between iron and lipid homeostasis [159,160,161,162] confirmed that the two pathways might share more than expected. Accordingly, by extensive investigation of common iron-regulating gene variants, a new mechanism of gene-gene interaction between iron genes and genes involved in the cholesterol metabolism by *APOE*-*HFE* has been proposed, paving the way to translational pharmacogenetic studies focused on the optimal tune of brain iron burden [163]. In this line, it has been found that also among other common cognitive diseases, males showed significantly higher peripheral iron levels compared to females. Conversely, among AD patients, females reached males levels, suggesting that the *APOE*-gene might be the key determinant in the processes of iron deposit in the brain, thus differently contribute to cognitive decline in the two sexes.

Fluctuations of oestrogen levels during perimenopause to menopause transition have been also suggested to be responsible for the increased AD risk experienced by menopausal females [164]. In this regard, different *APOE* genotypes have been reported to be differently responsive to oestrogens replacement treatment. Females carrying the *APOE*-ε4 allele showed the worst cognitive decline compared to untreated *APOE*-ε4 females, while *APOE*-ε2/3 females reported clinical improvements during oestrogen replacement therapy [165,166]. Accordingly, it has been recently suggested that different mechanisms, ranging from interactions between oestrogen receptors and ApoE to metabolic changes mediated by oestrogens might differentially affect *APOE*-carrier females [167]. Overall, oestrogen levels and oestrogen-based replacement therapies (including gender-transition therapies) will need to be carefully considered in the light of the *APOE* carrier status to prevent undesired brain disorders and cognitive decline. 

Although accumulation/deposition of amyloid-beta (Aβ) peptides and neurofibrillary tangles are the main hallmarks of AD, neuroinflammatory markers are receiving growing attention as direct mediators of the disease and as potential modulatory factors able to synergize with Aβ pathways in driving the disease progression [168].

In a recent work, Belloy and colleagues addressed the overall role of *APOE*4 in AD, concluding that its pleiotropic nature asks for integrated and synergistic studies, perhaps on extreme clinical phenotypes, contextually addressing neurologic, cardiovascular, and lipid driven inflammatory traits of the disease [150]. More efforts to better identify key pathways and molecular mechanisms underlying onset and progression of cognitive decline should be made also in the view of the ongoing ageing process of the worldwide population. The observation that dementia and CVD share common metabolic risk factors including inflammation, oxidative stress and lipids, affecting in turn both brain and myocardium [158,169,170,171,172], encouraged proteomic studies aimed at recognizing sex differences of the molecular signature of these complex diseases [173,174,175,176]. In conclusion, the comprehension of the molecular bases of the observed sex-differences in neurological disorders would improve by including both female and male animals in preclinical studies focused on brain OMICS change investigations [177]. The recognition that biological and genetic factors may be responsible for sex-related increased risk does not make dementia or AD a sex-specific disease but rather ask for new sex/gender oriented investigations and pharmacological studies.

## 5. Sex Disparity in Bone Homeostasis Diseases

A large number of studies have considered pathologies affecting bone, which are numerous and extremely heterogeneous. Bone is a highly dynamic tissue, constantly undergoing to catabolic and anabolic processes to maintain its flexibility and adapt to the demands of the organism for growth, mechanical loading and mineral balance [178]. Bone homeostasis is due to the opposite and complementary action of bone-forming cells (osteoblasts, OBs) and bone-resorbing cells (osteoclasts, OCs). Their synergy is implemented in a functional anatomic structure known as the basic multicellular unit (BMU) [179,180]. A considerable body of literature describes the effects of imbalances in the formation or resorption of bone, which may give rise to various diseases characterized by different levels of bone-remodelling cycle impairment, like osteoporosis, Paget’s disease and osteopetrosis [178,181]. The knowledge on the mechanisms underlying the formation and maintenance of bone is rapidly increasing, as well as the development of target therapeutic strategies against bone pathologies and skeletal degeneration [178,182,183]. However, extensive investigations are hampered by the limited accessibility of bone tissue, its mineralized nature, as well as by the complexity of the molecular aspects of bone turnover processes. As a result, not much work has been done to explore the role of sex/gender in the pathophysiology, diagnosis, prognosis, and treatment of bone diseases. Many critical issues remain open and further research is needed to address emerging new challenges in this field, and to identify relevant therapeutic targets.

Currently, the most relevant approach to this complex thematic considers the numerous variables that may affect the physio-pathological bone microenvironment, namely:

- the heterogeneity of the cell population: osteoclasts, osteoblasts, and osteocytes are the primary cells responsible for bone remodelling, yet other cells play a significant role, included their progeny, hematopoietic stem cells (HSC), cells of the immune system, mesenchymal stromal cells (MSC), adipocytes, endothelial cells and cells of the perivascular niche [178,184];
- the exposure over time to different combinations and doses of soluble molecules (nutrients and hormones) [178,183,185,186];
- the differences in the control of energy homeostasis [183,187];
- the signals that influence the MSC differentiation, not only in the osteoblasts, but also in the adipocytes, chondrocytes, and muscle cells [187,188,189];
- the cellular adaption to mechanical signals [190].

This complex picture is further complicated since all these variables are influenced by the age, but also by sex/gender. Structural deterioration of bone tissue may be due to loss of bone mass (osteopenia, osteoporosis) [191,192,193], or to an abnormal bone density increase (osteopetrosis, Paget’s disease, poisoning) [194,195]. Both occurrences, for different reasons (genetic, metabolic, primary, secondary, etc.), lead to bone fragility and to an increased risk of fractures in females as in males [196,197,198]. Sex hormones, oestrogens and androgens, influence the growth and the maintenance of the bones and are responsible for sexual dimorphisms [178,199]. An extensive crosstalk between sex steroids and growth factors such as IGF-1 and GH has been described, as well as their participation in the transduction of mechanical signals in bone [200,201,202]. A decline in the circulating levels of these hormones leads to loss of bone mass and functionality [200]. Qualitative and quantitative deficiencies drive the development of osteoporosis, which is the most common pathology affecting the bone [191,192,193]. This condition is correlated to physiological aging, being prevalent in particular in post-menopausal women [191,203], but can also occur as a consequence of pharmacologic treatment [197,203], or of feminizing hormone therapy in transsexual men [204,205]. Often mistakenly considered a female disease, osteoporotic fractures affect one in five men over the age of 50, and this number is set to increase dramatically as people around the world are ageing [198]. Evidence recently proposed shows that men are becoming the “weaker sex” in terms of death and disability caused by osteoporosis; the health-care systems simply disregard their bone health [198]. To date, osteoporosis in men remains underdiagnosed and underestimated. Efforts to reduce sex-disparities regarding osteoporosis are becoming imperative not only to improve the quality of life of the male sex, but also of the transsexual adults [198,204,205].

A further challenge is represented by exploring the functional interactions between bone-related diseases and molecular biomarkers and networks. Accordingly, omics studies (transcriptomics, epigenomics, proteomics and metabolomics) performed on the human bone may reveal valuable sex distinctive changes [206,207]. Thus, scientists and physicians who deal with skeletal disorders cannot ignore anymore sex/gender-omics in both preclinical research and medical clinical trials. 

## 6. Sex Disparity in Osteoporosis Risk and Prevention

Sex is a known key clinical risk factor for involutional osteoporosis, second in importance only to aging. Oestrogen dependent menopausal bone loss is usually more critical in the spine rather than in the hip and total body. Trabecular bone is lost by perforation with complete loss of trabeculae leading to increased trabecular spacing; cortical bone is lost as a result of unbalanced intracortical remodelling, which increases cortical microporosity, enlarge the intracortical canal surfaces and reduce cortical bone accrual at the periosteum. With advancing age, trabecular bone loss declines because the trabeculae disappear and the cortical bone loss become predominant. 

In men, the gradual reduction in levels of sexual steroid hormones leads to a bone loss characterized by reduced bone formation associated with modest increases in bone resorption, which causes a progressive thinning of the trabeculae. Cortical bone loss in man is delayed over time compared to women, determining a smaller microstructural deterioration and, consequently, lower reduction in bone mineral density and a higher fracture risk at an older age [208,209]. 

**Figure 6:**
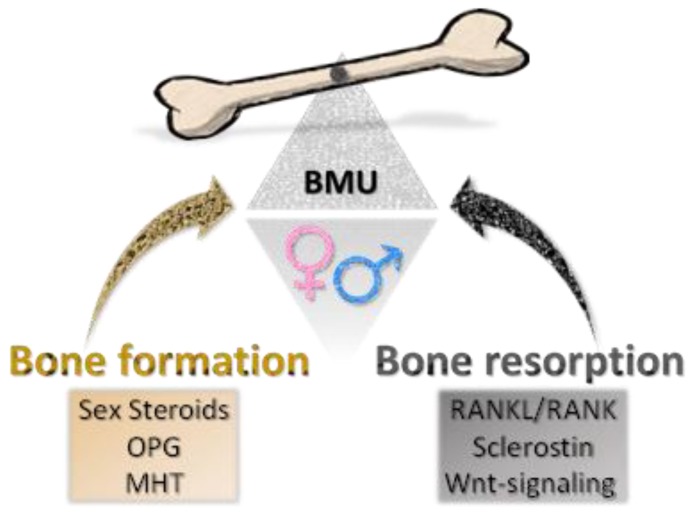
Different actors and effects on bone formation/resorption balance according to different sexes. BMU: Basic Multicellular Unit; OPG: Osteoprotegerin; MHT: Menopausal Hormone Therapy; RANKL: Receptor activator of nuclear factor kappa-Β ligand; RANK: Receptor activator of nuclear factor kappa-Β.

Different cell types and mechanisms mediate the effects of sex steroids on cancellous and cortical bone. As previously reported, oestrogens exert anti-remodelling effects in different ways by decreasing osteoclastogenesis and inducing apoptosis in osteoclastic cells. Accordingly, they maintain bone formation organizing commitment and differentiation of osteoblastic cells, preventing their apoptosis and modulating their activity. The anti-resorptive action of oestrogens is also due to the induction of genes expression and synthesis of OPG (osteoprotegerin), a determinant factor of the RANKL/RANK system. Furthermore, oestrogens reduce the apoptosis of the osteocyte (Figure 6). Recent studies have shown that oestradiol levels are inversely associated with serum levels of sclerostin, a key inhibitor of the Wnt-signalling produced by osteocytes, and that oestrogen deficiency may contribute to the development of osteoporosis by decreasing the sensitivity of osteocytes to mechanical loading [210]. Finally, the oestrogen ability to improve calcium balance by regulating enterocyte calcium influx through ER has also been shown [211].Epidemiological evidences suggest that bioavailable oestradiol (E2) and/or sex hormone binding globulin (SHBG), but not serum testosterone levels are associated with bone loss in men. Results from study of murine models with cell-specific deletion of ER and androgen receptor (AR) demonstrate that there are many cell types that mediate androgens effects on the bone in males. Yet, while the oestrogenic anti-resorptive effect on cancellous bone is a direct one with an action on osteoclasts, androgens play their anti-resorptive effect indirectly through osteoblasts and osteocytes [212].

Since the main risk factor for fragility fractures in postmenopausal women is the bone loss due to oestrogen deficiency, the menopausal hormone therapy (MHT) is a rational therapy for prevention of osteoporosis [213]. Convergent data from research studies and clinical trials indicate that MHT, both in the form of combined oestrogen and progesterone or oestrogen alone, normalizes bone turnover and preserves bone mineral density (BMD) at all skeletal sites, significantly decreasing vertebral and non-vertebral fractures in long-term treated subjects [214]. This suggests that MHT may, at least in part, fill the gender gap in osteoporosis risk in particular if MHT is starting early after menopause. Oestrogen therapy, by influencing the lifespan of osteoblasts and osteoclasts, indeed, may reverse the oestradiol dependent component of the remodelling imbalance. It is to emphasize that remodelling imbalance occurring around the ages of 45–50 years, produce irreversible deficits in bone volume, microstructural deterioration and bone fragility. Starting an anti-resorptive therapy in late postmenopause doesn’t reverse existing microstructural deterioration and doesn’t abolish bone fragility [215]. Despite the concern emerging from the large, long-term RCT Women’s Health Initiative (WHI) study about the safety of hormonal replacement treatment, post hoc subgroup analyses, stratifying women according to their age and time since menopause, have allowed to better understand the relationship between MHT and cardio-vascular risk. Timing of MHT initiation was shown to be a critical factor for the development of cardio-vascular diseases. According to the “timing hypothesis”, healthy symptomatic women who initiated MHT when aged younger than 60 years, or who were within 10 years after last menstrual period, have demonstrated a reduction in both coronary heart disease risk and all-cause mortality [216,217,218]. Moreover, the term ‘MHT’ does not identify a single treatment but different forms, doses and ways of administration of hormonal replacement therapy. The concept of tailored MHT includes the choice of an effective and appropriate dose, type, and regimen that differs according to age, clinical characteristics and goals to achieve for each woman. The choice of the right progestin is critical as it can make the difference in long-term outcomes [219]. Lastly, there is an unmet need for providing and approving testosterone treatments specific for women, formulated with the aim of approximating physiological testosterone concentrations for premenopausal women [220]. Nowadays just a few studies have evaluated the musculoskeletal effects of testosterone. The available data do not support an effect of testosterone treatment on BMD at the spine or femoral neck at 12 months; nevertheless, there is a need for clinical trials to evaluate the impact of testosterone treatment on musculoskeletal tissues. The main indication for MHT is the presence of vasomotor symptoms (VMS), which have been clearly associated with the risk of cardio-vascular disease, osteoporosis and cognitive decline, as a clinical marker of susceptibility to oestrogen deprivation. Thus, moderate to severe vasomotor symptoms can be seen as independent predictors of low BMD and fracture risk [219]. By treating younger, healthy but symptomatic, postmenopausal women, we target the population that can mostly profit from tailored MHT, with clear evidence of a definite long-term benefit. Fear of breast cancer risk had probably the greatest negative effect on the use of MHT. Recently, *The Lancet* published a study by the Collaborative Group on Hormonal Factors in Breast Cancer [221] in which data from 58 studies were combined using a nested case-control study design to focus on the long-term effects of MHT. They concluded that women who initiated MHT shortly after menopause had a significantly increased risk of invasive breast cancer compared with never users. As pointed out by the International Menopause Society (IMS) press release [222] it is important to emphasize that potential breast cancer risk is one component of the benefits/risk analysis of MHT use for an individual woman, and that this analysis needs to include symptoms severity and potential beneficial effects of MHT on bone, cardiovascular health and sexual life. Anyway, it is extremely important to note that this paper does not clarify the impact of currently used hormonal therapy dosage and schemes on breast cancer risk [221]. Consequently, it will be important to perform studies about the long-term risk-benefit ratio of new MHT formulations, doses and routes of administration. Virtually old hormonal formulations and doses, which are known to be responsible of adverse breast effects, can no longer be prescribed. Hence, individualization of care is necessary in order to achieve a safe personalized menopausal hormonal treatment to counteract the deleterious effects of postmenopausal oestrogen deficiency on women’s bone health.

## 7. Sex Disparity in Morbidity/Mortality of Infectious Diseases

Infectious diseases have accompanied the human being likely from the beginning of its existence. The presence of some kinds of infections may have been enhanced through past events and cultural transitions, like those of the Neolithic Era, which brought humans and animals in close contact, possibly fostering the interspecific spread of transmissible diseases [223]. Some trace of animal-to-human transmission has been retrieved in ancient skeletons of victims [224]. The condition of crowded settlements that can promote epidemic spread of communicable diseases among humans, including through vectors [225,226], may have arisen during the Neolithic as well, about 12,000 years before present.

In evolutionary terms, the long-lasting and repeated contact between humans and pathogens may have induced co-evolutionary processes, which have modulated the co-existence of the two systems, if not the eradication either of the pathogen or of human groups [223]. In the long time of coexistence with their hosts, pathogens should have faced many sources of stress, including the biological variability of the hosts, their different nutritional conditions, body sizes, immune status and physiology [227].

Co-existence may have modulated genomic variability in humans as well, particularly in those genes distributed on autosomal chromosomes, which, under selective pressure, may have developed higher incidence of protecting genetic variants. These genes have gained some attention [228]. Yet, the major difference among mammalian individuals, which persists at least from 180 millions of years [229], the difference between the two sexes, is not yet taken in account in many studies, despite the increasing evidence that males and females may be differently affected, in terms of morbidity and/or mortality, by infectious diseases. Notwithstanding, the first study in this direction is quite old [230]. 

On one side, the differences among sexes in terms of susceptibility, morbidity and mortality can be due to distinct cultural behaviours, thus be related to their gender more than to their biological sex. Females and males differ in general for their exposition to pathogens due to their gender-specific activities [231]. Men tend to be more often outside home, for instance for hunting in the wood, where they could meet Ebola- [231] or otherwise infected animals, or being working on ships or docs dealing with plague- [232] or otherwise infected goods. Women, in the majority of the societies, “are more likely than men to be caregivers for the sick in both health-care settings and at home” [231]. Zoonosis (i.e., diseases of animals and livestock), can occur more often in that gender traditionally providing care for animals [231]. Finally, gender differentiated access to health care in different societies may also drive gender-based bias in the exposure to infectious diseases [231]. In India for example, ill boys were observed to be brought to professional care significantly sooner than ill girls [233], whereas in Bangladesh, the time of admission at the hospital for children with diarrhoea differed significantly in favour of boys [234].

Still, gender bias and behaviours cannot account alone for the disparities observed between sexes in contact with communicable diseases. Biological differences play a comparably important role. Dissimilarities may be observed at any biological level, yet the chief difference observed between males and females consists in the broad influence that sex-hormones have demonstrated in the host immune response [90,235,236]. The immune response of females against pathogens seems to be stronger in respect to that of males [90,237,238]. Females tend to clear infections faster and show less probability of infection persistence [237,239,240], due to oestrogen working as an activator of immunity, whereas testosterone as a suppressor [241]. On the other hand, possibly for the same reason, females are more prone to develop autoimmunity [237,239,240,242]. 

At the gene expression level, sexual dimorphism mainly involves the X chromosome, which contains high proportions of immune-related genes and regulatory elements producing different innate and adaptive immune responses to infections [243]. The effects of the X chromosome are likely independent from those of sex hormones, despite both systems influence the immune response [244]. Having two X chromosomes, and a complex regulating mechanism, X-heterozygous females can activate a mosaic advantage, with the half of the cells expressing the wild type and the other half the variant, compared to males having only one X chromosome [243]. 

Thus, and as a rule, men experience greater susceptibility to infectious diseases, as well as severity of infection, when compared to women [239,240,242,245,246,247]. The progression to active tuberculosis after infection with Mycobaterium tuberculosis is 1.5-times more likely in men than in women [248,249,250]. Studies devoted to clarify the genetic mechanism responsive of the sex bias have given contradictory results, possibly because the outcomes have been concealed by the effects of environmental factors, as well as by the presence of different strains of the pathogen (reviewed in [243]). The region Xq26 was identified as susceptible to tuberculosis by the first genome-wide linkage analysis, which anyway did not consider sex bias [243,251]. Amoebic liver abscess (ALA), caused by the intestinal protozoan parasite Entamoeba histolytica, represents an example of a parasitic disease with a bias toward males (reviewed in [252]). Despite ethnic or cultural background, more than 80% of ALA cases worldwide occurs in adult men, with an increasing risk from puberty to the highest during 30–50 years of age (male-to-female ratio of approximately 7:1). A correlation of ALA’s occurrence with the increase in levels of testosterone was suggested (reviewed in [252]). Malaria reports from a hypo-endemic area of India, has showed as well a bias in infection by Plasmodium vivax and *P. falciparum* infections towards pubertal males and a peak during 30-45 years of age, when compared with females of the same age [252,253]. Men are also more frequently infected by leishmaniosis than women ([254,255,256,257] reviewed in [252]), with a prevalence of American cutaneous leishmaniosis after the fifteenth year of age [252,256]. Cryptococcus neoformans causing meningitis has showed a distinct biological behaviour towards male hosts, due to differential macrophages’ activity and differential expression of the pathogen virulence genes in the two sexes [258]. A recent study [240], has proposed an alternative, better, additional, explanation for sex-bias in infectious diseases, based on an evolutionary perspective. Starting from a pathogen-centred viewpoint (instead of a host-centred), the authors formulated an epidemiological model to test the hypothesis of a sex-preferential behaviour of pathogens in favour of women, due to their unique ability to give rise to vertical transmission through pregnancy, birth and/or breast-feeding, in addition to the traditional horizontal spread. By developing sex-selective virulence in favour of women, pathogens would enhance their possibility of dissemination, in particular during prolonged breast-feeding. The model confirmed that pathogens with a mixed (horizontal and vertical) transmission evolve to be less virulent, thus providing an explanation for a lower virulence in females than in males [240]. 

Nevertheless, during pregnancy there are important changes in the immune system of women, which are not yet well understood [231]. Some diseases are demonstrated to be particularly virulent during pregnancy or adversely affect the foetus or breastfeeding baby [231]. Among non-immune women, *P. falciparum* malaria is often more severe during pregnancy than at other times developing a much higher parasitaemia. Women with leprosy have a greater risk of nerve deterioration during pregnancy and lactation than at other times [231].

Yet, in some cases, even if not pregnant or breastfeeding, females are more prone to be affected by infections and die than males. Mortality from measles and whooping cough is greater in females, albeit rates of measles infection are broadly similar for males and females [231,259]. Urinal trait infections exhibit higher susceptibility and prevalence in women, but higher severity in men (reviewed in [260]). This effect cannot only be due to anatomical differences of the urinal trait [261], since the main sex-specific dissimilarities in the incidences of urinal trait infections has been observed in the life period with the highest levels of sex-hormones [260]. 

In particular, for epidemic-prone infectious diseases, WHO observed: “Despite the potential importance of differences in sex and gender for the transmission, course and outcome of some infectious diseases, little has been written about the implications of sex and gender for the surveillance of and response to outbreaks, especially for diseases that are not sexually transmitted”. The WHO attributed this phenomenon to the lack of interaction among different disciplines, like “epidemiology, medical and biological sciences, social sciences and demography” [231]. They offered to put different findings together and translate them “into recommendations for WHO operational responses to outbreaks” [231]. More than a decade later, apparently, only few researchers have acted on these recommendations.

In conclusion, although evidence is mounting that not only sexual but also other kinds of infectious diseases differently act in females and males, researchers do not always distinguish between sexes and the treatment provided to patients of both sexes tend to be the same [237,239,240]. One of the reason for this gap is the lack of knowledge and information about sex-specific distinctions in morbidity and mortality of many infectious pathologies [240,262]. Even in the case of epidemic-prone diseases, more detailed information in the reports on patients would lead to a better understanding of sex bias in infection and lethality, thus helping in the identification of susceptible groups and development of appropriate responses to avoid or stop outbreaks [231]. In the research field, the differential consideration of data might help in a better understanding of the immune response mechanisms involved and their evolution over time, which is the first step on the pathway of setting a precise pharmacological defence.

## 8. Sex Disparity in Pain Threshold and Feelings

Pain is a major healthcare problem worldwide that most affect women. Acute pain can reasonably be considered a symptom of disease or injury, whilst chronic and recurrent pain needs specific medical and pharmacological care [263]. This complex experience, which shows significant differences with respect to gender, derives from the integration between physical, psychic and socio-cultural components. According to a survey of over 85 thousand adults in 17 countries around the world, chronic pain symptoms of any kind afflict 45% of women, compared to 31% of men [264]. In recent decades, the results of many studies examining the pathogenesis of neuropathic pain and its prevention and treatment strategies have confirmed that the pain threshold is sex-specific and females appear to feel pain more intensely with a lower threshold than men [265]. Some studies have shown that health medical staff treat a disease more seriously when a man reports it, even if the other people present the same symptoms. Accordingly, prescription of opioids is mainly aimed at men, while for women the psychological components of the disease are more considered, and gender differences could be attributable to cultural influences [266]. Boys and girls grow up with different outlooks on pain: girls often feel free to cry over small injuries, while boys feel extra pressure to hold back the tears [267]. Unfortunately for women, an emotional response can make a painful situation worse: women are more likely than men to develop anxiety or depression that can increase feelings of pain and the risk of disability [268]. 

The gender gap in pain threshold and feeling is probably linked to biological and genetics differences in male and female bodies and to their capability to respond in a different way to pain. The knowledge of these differences can shed light on the basic nature of pain and lead to improved treatments for all patients [269]. Oestrogen receptors are distributed in many pain-related regions in both the central and the peripheral nervous systems, so that oestrogens can influence the generation and transmission of pain [270]. A connection has been observed between oestrogens and the N-methyl-d-aspartic acid (NMDA) receptor 1, which is able to bind an excitatory neurotransmitter, glutamate, involved in the generation, transmission and maintenance of various types of pain [271]. In particular, NMDA receptors play a primary role in the transmission and regulation of information regarding neuropathic pain and their expression is increased by the presence of oestrogens [272,273]. It has been also observed that gonadal oestradiol (E2) alters the sensation of pain through upregulation of vanilloid receptors 1 (TRPV1) that plays a critical role in triggering pain. An increase in progesterone seems to determine a down-regulation of TRPV1 receptors in the plasma membrane of sensory neurons, decreasing the perception of pain under physiological conditions like pregnancy [274]. In contrast, testosterone has been shown to play a key role in inhibiting the expression of TRPV1 at the sensory ganglion level in a model of chronic inflammatory pain induced in rats, thus confirming the presence of sex differences for neuropathic and/or chronic pain in both animals and humans. 

**Figure 7:**
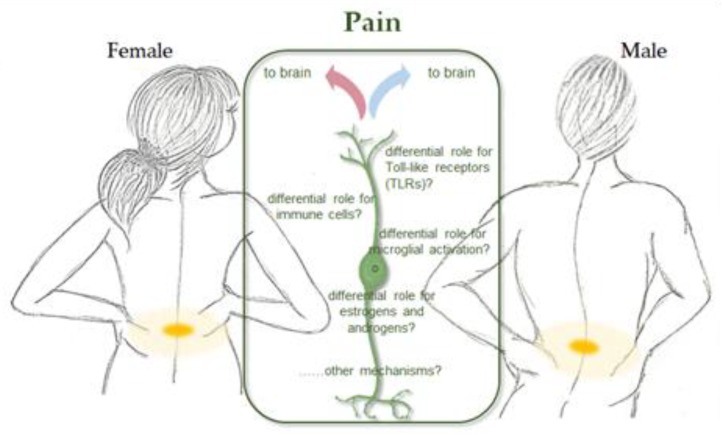
Hypothesis of various cellular mechanisms involving pain transmission in females and males.

Preclinical studies should be performed in male and female animals, and at different ages, because hormonal cycles can also influence the results, whereas the nerve pathways of pain are different [275]. Female rodents have a lower pain threshold in experimental models of hot thermal, chemical, inflammatory and mechanical nociception [276]. As a consequence, it is better not just to assume that data obtained from experiments conducted on male animals can be generalized to both sexes, and not to transfer the results of clinical trials from male to female [277]. Males and females do not have the same bodies and there are significant differences in pharmacokinetics and pharmacodynamics which could modify drug doses, therapeutic effect and toxicity [278]. Regarding the pain transmission and the cellular involvement, the microglia appears to have a prominent role in the pain for males, but not for females, who are more sensitive to the control of T-lymphocytes, a type of cell whose role is known in sensitization to pain [279]. Clinical research is fundamental to verify whether the cellular mechanisms of pain, as well as the involvement of cells and/or microglia, are different between men and women in a similar way to those found in the animal models (Figure 7). It is evident that hormones influence perception of pain: oestrogens mitigate pain with concentration-dependent mechanisms, whereas testosterone reduces the sensitivity to chronic pain [280]. Pain responses seem to change during life depending on hormonal variability: at puberty, the perception of pain increases in women, while sex differences in chronic pain rates disappear in menopause [281]. The gonadal hormones affect the incidence of pain, as it was observed in transsexuals who received cross sex hormones to develop and maintain somatic characteristics of the opposite sex [282]. The male to female transsexuals showed increased pain sensitivity with oestrogen treatment, whilst the female to male transsexuals treated with testosterone reported a significant reduction of the chronic pain [283].

The pharmaceutical market offers the same pain-relieving drugs to everyone, even though sex-differentiated pharmacological treatments could work better. Numerous difficulties have arisen in identifying a pharmacological dosage according to sex, due to the lack of female subjects in almost all clinical studies. In general, women in their reproductive age are excluded from the trials due to rapid hormonal changes and possible pregnancy [284]. As far as the analgesic therapy is concerned, the response is different in males and females, since men are more sensitive to the use of non-steroidal anti-inflammatory drugs, while morphine shows greater efficacy in women [285]. Several major factors can explain sex-differences in the modulation of pain with opioids, such as the neuro-anatomical organization and the neuro-physiological characteristics of the descending inhibitory circuit, whilst no sex-difference is known in the plasma concentration of morphine and/or its metabolites [286]. A series of studies carried out on post-operative pain revealed that male patients consumed morphine doses 2.4-times higher than women, confirming that women are more sensitive than men to morphine [287]. The hepatic metabolism of the opiates is different for men and women, and these differences depend on the presence of sex hormones in circulation and on their influence on pharmacokinetics, pharmacodynamics and pharmacogenetics [269,288].

The awareness that the biological basis of pain differs between males and females raises important questions in experimental research. Recommendations may be required to improve the regulation of the drugs with regard to sex and gender. The development of potential drugs with fewer negative side effects and more favourable pharmacological properties for both sexes could make an important contribution to gender medicine and pharmacology, as well as to the development of personalized health care to both women and men.

## 9. Sex/Gender Disparity in the OMICs Era: Sex-Omics and Gender-Omics

Omics-sciences investigate the role of genes, proteins and metabolic pathways involved in disease susceptibility with the aim to improve diagnosis, prognosis and novel drug design. Besides to genomics, proteomics and metabolomics, new omics have been proposed and referred as ”sex-omics” and “gender-omics” aimed at investigating sex/gender specific aspects in biomedical sciences [289,290,291]. Whilst sex-omics is easier to be faced, knowing the sex-related side of any pathological condition, gender-omics has a more complex and multifaceted nature that makes it difficult to be approached.

Great progresses have been made in the milestones studies of Arthur P. Arnold on the “sexome”, defined as the sum of sex-biased effects on gene networks and cell systems [292]. Several pathological conditions differently affect females and males, and this is an extraordinary occasion to find out risk and protection factors differently expressed in the two sexes [293]. In brief, sex-biasing factors include two X-chromosomes and ovarian secretions in females, and a single X chromosome, the Y-chromosome and testicular secretions in males. Although, it might be considered a reducing consideration, this model has dramatic biologic effects, as shown in mouse tissues not directly involved in reproduction, where transcriptome analyses showed sex differences in 72% of the liver genes and in 68% of the adipose [20,292]. Of note, different sex-specific evolutionary processes have selected the two sex chromosomes, and whilst the Y-chromosome would be expected to carry male-benefiting genes, the X-chromosome should contain both male- and female-benefiting genes. Accordingly, sex differences in development and behavior are necessarily ascribed to those genes, which are differentially expressed in male and female cells, and selected for sex-specific roles. Considering the brain as a sexually dimorphic organ molded by sex-specific selection pressures, genes on the sex chromosomes may probably determine gender (i.e., sexually dimorphic phenotype) by inducing sex differences and sex-specific effects on XX and XY brain cells [294], although the influence of the environment in any individual life should not be disregarded.

Scientific developments in biomedical fields have identified the underlying causes of many inherited or acquired human diseases that can be potentially faced by precision medicine with the aim to have treatments tailored to each patient. Reductionism in biomedical research has resulted in the identification of the exact mutation in the genome, a defective molecule or a particular cell phenotype responsible for that human disease counteracted by targeted therapies. To have a more complete understanding and comprehensive explanation of the molecular pathogenesis of the disease mechanisms, omics-sciences can greatly help in the recognition of intricate candidate pathways in those complex diseases in which the one-gene-defect model or one disease-one assay-one drug principle is not enough. Accordingly, gender medicine is a promising field in which multi-omics approaches could provide significant input in terms of personalized medicine: in other words, sexomics and genderomics.

Important differences between males and females, considered intrinsic natural differences, are already recognizable at the stage of the in utero zygote implantation and endure during prenatal development, childhood and adulthood. Omics technologies, including genomics, epigenomics, transcriptomics, proteomics and metabolomics, can be successfully applied to firstly investigate those reasons responsible for the sex- and gender-related diversities in the pathophysiology and etiopathogenesis of complex multifactorial and multigenic diseases [32,33]. Personalized-omics will transform traditional medicine from symptoms-oriented diagnosis and treatment towards disease prevention and very early diagnosis.

By considering symptoms-oriented diagnosis, we were focused only on late symptoms of a manifest disease, neglecting important preclinical signs and specific symptoms useful as discriminant in a personalized medicine approach. Also considering the same disease, male and female may have completely different preclinical signs and/or disease progression and prognosis, as during the acute phases of myocardial infarction. In addition, female/woman-specific risk factors, as body fat distribution, or specific prognostic markers have been identified by proteomics investigations [33]. Accordingly, personalized omics-approaches can be strongly useful in earlier recognition of any informative data in a broad range of pathologies.

Sex differences and sexual dimorphism mainly involves the X-chromosome, which contains high proportions of immune-related genes and regulatory elements, affecting in turn the different susceptibility to some complex diseases, such as certain kind of cancer, autoimmunity, infections, and bone homeostasis related disorders, in which noticeable sex-differences exist [295,296]. The rapid technological progress and the excellent bioinformatics methods currently available yielded us a large volumes of data needing sophisticate computational analyses for acquisition, storage, handling and translation in an applicative personalized diagnostic/prognostic protocol [297].

**Figure 8:**
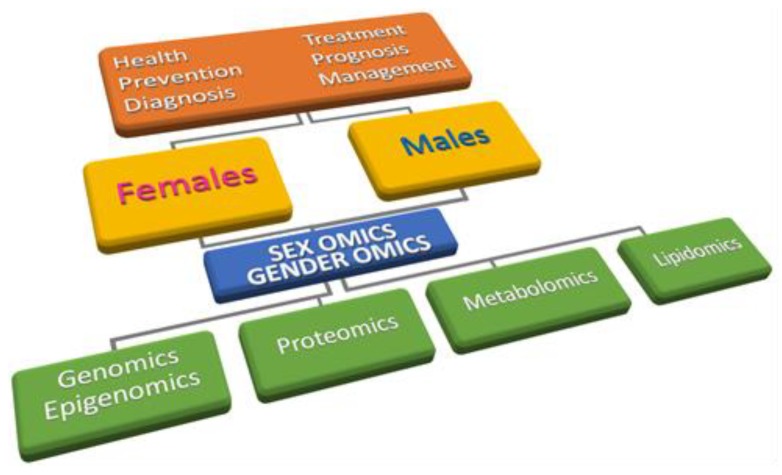
Proposed sex-omics/gender-omics strategy for research and clinical approach to diseases.

By using multi-omics approaches, to integrate information about gene expression and protein level and composition, we will have a wide-ranging comprehension on how sex and gender affects a particular disease or pathological condition (Figure 8). This novel approach, though complex and difficult to obtain, is a fruitful tactic to make major progress and contribution in a personalized gender medicine [298].

Finally, omics sciences if sex and gender-oriented, will also help other important auxiliary health-related disciplines, such as those involved in food-sciences and nutrition, where efforts to recognize personalized diets in relationship to health is the first step in preventive medicine [299]. One extraordinary field in which this could be applied is the multivitamin supplement (e.g., folic acid) during the pre- and peri-conception period, not universally considered safe for every mother in absence of a detailed genomic analysis [300,301,302]. Finally, if part of these advances will be accomplished, this will significantly improve the global health at any age and stage and for any sex and gender cutting down health care costs of the Public Health Systems. Everyone must make their own effort, including scientists, governments, pharmaceutical companies, patient associations and scientific journals working together to ensure the success of this epochal transformation.

## 10. Conclusions

There is a growing body of evidence suggesting that there are sex and gender differences between clinical manifestations, disease progression, treatment efficacy and prognosis in several common or complex pathologic conditions. A number of faults and underestimations have been performed in the past by not properly considering that different sexes have different genetic, biological and psychological features, which greatly influence the natural disease course, symptoms and treatment response. As personalized medicine and pharmacogenetics are taking hold in the common medical procedures, gender medicine and omics-approaches equally have to become part of the clinical practice. The present review article dealt with sex-specific manifestations of classical and less known pathologies and situations in terms of impasse in the diagnostic, prognostic and treatment options we have at disposal in the clinical practice. Finally, there is a number of exciting challenges to face in the years to come, such as the inclusion of gender in both preclinical and clinical studies to understand any unrevealed male and female difference and intersection, with the final aim to achieve a fully inclusive personalized medicine:

*“It is also important to note that the study of sex/gender differences benefits men as much as it benefits women. Therefore, when we fail to routinely consider the impact of sex/gender in research, we are leaving everyone’s health to chance”.*

[298]
